# Lateral fluid-percussion injury leads to pituitary atrophy in rats

**DOI:** 10.1038/s41598-019-48404-w

**Published:** 2019-08-14

**Authors:** Mehwish Anwer, Riikka Immonen, Nick M. E. A. Hayward, Xavier Ekolle Ndode-Ekane, Noora Puhakka, Olli Gröhn, Asla Pitkänen

**Affiliations:** 0000 0001 0726 2490 grid.9668.1 A. I. Virtanen Institute for Molecular Sciences, University of Eastern Finland, Kuopio, Finland

**Keywords:** Experimental models of disease, Epilepsy, Prognostic markers

## Abstract

Traumatic brain injury (TBI) causes neuroendocrine dysregulation in up to 40% of humans, which is related to impaired function of the hypothalamo-hypophyseal axis and contributes to TBI-related co-morbidities. Our objective was to investigate whether hypophyseal atrophy can be recapitulated in rat lateral fluid-percussion injury model of human TBI. High-resolution structural magnetic resonance images (MRI) were acquired from rats at 2 days and 5 months post-TBI. To measure the lobe-specific volumetric changes, manganese-enhanced MRI (MEMRI) scans were acquired from rats at 8 months post-TBI, which also underwent the pentylenetetrazol (PTZ) seizure susceptibility and Morris water-maze spatial memory tests. MRI revealed no differences in the total hypophyseal volume between TBI and controls at 2 days, 5 months or 8 months post-TBI. Surprisingly, MEMRI at 8 months post-TBI indicated a 17% reduction in neurohypophyseal volume in the TBI group as compared to controls (1.04 ± 0.05 mm^3^ vs 1.25 ± 0.05 mm^3^, p < 0.05). Moreover, neurohypophyseal volume inversely correlated with the number of PTZ-induced epileptiform discharges and the mean latency to platform in the Morris water-maze test. Our data demonstrate that TBI leads to neurohypophyseal lobe-specific atrophy and may serve as a prognostic biomarker for post-TBI outcome.

## Introduction

Globally, an estimated 69 million people experience traumatic brain Injury (TBI) each year^1^. Patients suffer from neurobiological, psychological and social consequences of injury^2^. Furthermore, TBI causes 15–20% of acquired epilepsy in the general population and 5% of all epilepsies^3,4^. In addition to initial focal and systemic changes, TBI can also induce long-term global alterations in the neuroendocrine system^5^. About 25–40% of moderate and severe TBI patients develop chronic hypopituitarism^6–9^. The hypothalamo-pituitary axis modulates neuroendocrine and autonomic functions, and its atrophy can result in chronic pituitary dysfunction^10^. For instance, abnormal vasopressin release from the posterior lobe after TBI resulted in diabetes insipidus (DI)^11^. Impaired hypothalamo-pituitary-adrenal (HPA) axis and growth hormone deficiency are the most commonly reported endocrine dysfunctions of the anterior lobe after TBI, and have been extensively linked to depression, poor quality of life and rehabilitation outcomes^12,13^. However, posttraumatic hypopituitarism may be underdiagnosed due to its delayed onset and overlapping signs and symptoms^13^. Both TBI and chronic hypopituitarism result in similar neurobehavioral complications including memory and concentration deficits, depression, anxiety, fatigue and loss of emotional wellbeing^14,15^, which suggests that untreated post-TBI endocrinopathy may contribute to the development of post-traumatic stress disorder (PTSD)^16^. Recent studies have also identified abnormalities of the HPA axis in patients with epilepsy^17^. Conversely, abnormal secretion of vasopressin due to pituitary dysfunction have resulted in hyponatremia, leading to seizures in humans^18^. The implications of HPA axis dysfunction in the generation of seizures and comorbidities in humans are yet to be explored.

Magnetic resonance imaging (MRI) serves as a versatile non-invasive neuroimaging method to study the spatio-temporal progression of disease in brain. MRI studies in patients with posttraumatic hypopituitarism identified hemorrhagic lesions and infarction in adenohypophysis and neurohypophysis^19^. However, only a few studies have employed MRI to study structural abnormalities in the hypophysis after experimental TBI in rodents^5,20,21^. Manganese-enhanced MRI (MEMRI) is an emerging method utilizing Mn^2+^ as a contrast agent for imaging functional neural circuits and the anatomy of the rodent brain *in vivo*^22^. Administration of MnCl_2_ systemically allows profound contrast enhancement in areas of the brain that lack the blood brain barrier, such as the pituitary gland. In addition, it has been shown that MEMRI differentiates the two pituitary lobes, the adenohypophysis and neurohypophysis, distinctively^23^.

In the present study, we investigated the structural changes in the hypophysis at acute and chronic time-points after lateral fluid percussion injury (FPI) in rats, using high-resolution structural MRI and manganese-enhanced MRI (MEMRI). We demonstrate that the neurohypophyseal volume is reduced after TBI, despite no detectable changes in total hypophyseal volume. Further, neurohypophyseal atrophy associated with increased seizure susceptibility and memory impairment.

## Materials and Methods

### Study design

This is a retrospective study reviewing the MRI scans from 2 cohorts of rats that received lateral fluid-percussion injury. In cohort 1, MRI scans were acquired at 2 days and 5 months post-TBI. The total hypophyseal volume was estimated in sagittal plane. In cohort 2, MnCl_2_-enhanced MRI (MEMRI) was acquired at 8 months post-TBI. The total hypophyseal volume was estimated both in coronal and sagittal planes. In addition to total hypophyseal volume, the adenohypophyseal and neurohypophyseal volumes were also estimated. In addition to MEMRI scans, seizure susceptibility (determined by pentylenetetrazol (PTZ) test) and hippocampus-dependent learning and memory (determined by Morris water maze test) were evaluated.

### Animals

Two cohorts of adult (age 12–14 weeks, 320–450 g) male Sprague-Dawley rats (cohort 1, n = 34, Harlan Laboratories, S.R.L., Italy; cohort 2, n = 19, Harlan Netherlands B.V., Horst, Netherlands (now Envigo Laboratories)) were used in this study. Animals were housed in a controlled environment (temperature 22 ± 1 °C, humidity 50–60%, light–dark cycle from 07.00 to 19.00 h) with free access to food and water. All animal procedures were approved by the Animal Ethics Committee of the Provincial Government of Southern Finland, and performed in accordance with the guidelines of the European Community Council Directives 2010/63/EU.

### Lateral fluid-percussion injury

Severe TBI (>2.8 atm) was induced using the lateral FPI model of injury as described previously^24–28^.

#### Cohort 1

TBI was induced in 14 rats using lateral FPI^24,25^. Animals were anesthetized using 5% isoflurane (room air as a carrier gas) and maintained with 1.9% isoflurane (Somnosuite # SS6069B, Kent Scientific). The animals were then placed in a stereotaxic frame (David Kopf Instruments, Tujunga, CA, USA), the skull was exposed and a circular craniectomy ($\varnothing$5 mm) was performed over the left parietal area midway between lambda and bregma, leaving the dura intact. Lateral FPI (impact severity 2.8–3.0 atm) was induced by connecting the rat to the fluid-percussion device (AmScien Instruments, Richmond, VA, USA). Sham operated animals (n = 5) went through the same procedures except the fluid-percussion injury.

#### Cohort 2

TBI was induced in 12 rats using lateral FPI^24,25^. Animals were anesthetized with a cocktail (6 ml/kg, i.p.) of sodium pentobarbital (58 mg/kg), chloral hydrate (60 mg/kg), magnesium sulfate (127.2 mg/kg), propylene glycol (42.8%), and absolute ethanol (11.6%). The animals were placed in a stereotaxic frame and craniectomy was performed, leaving the dura intact. Lateral FPI (impact severity 3.2–3.4 atm) was induced by connecting the rat to the fluid-percussion device (AmScien Instruments, Richmond, VA, USA). Sham operated animals (n = 7) went through the same procedures except the fluid-percussion injury.

### Magnetic resonance imaging

Rats were anesthetized using 1.5–2% isoflurane (carrier gas mixture of 70% N_2_ and 30%O_2_) and were secured in a holder using ear bars and a bite bar while anesthesia was delivered through a nose cone. Breath rate and body temperature were monitored.

#### Structural MRI (Cohort 1)

Animals were first scanned at 2 days (d) post-TBI and then a follow-up MRI scan of the same rats was done at 5 months post-TBI. MRI was performed with a 7T scanner (Bruker Pharmascan, Billerica, MA) operated with ParaVision 5.1 software. An actively decoupled quadrature resonator volume transmitter coil and a rat brain quadrature surface receiver coil (Rapid Biomedical, Germany) were used to acquire high resolution structural 3D MRI. A multi echo gradient echo (MGE) sequence with 66 ms repetition time, flip angle of 16 degrees, field of view 25.6 × 19.5 × 12.8 mm, matrix 160 × 122 × 80, 1 average, 120 dummy scans to obtain steady state, outer volume suppression and fat suppression was used. Thirteen echoes with echo time ranging from 2.7 ms to 43 ms with 3.1 ms interval were collected, and all 13 echo images were summed together. This resulted in T1/T2* mixed contrast and 160 × 160 × 160 µm^3^ spatial resolution.

#### MEMRI (Cohort 2)

At 8 months post-TBI, MRI was performed with a 4.7 T scanner (Magnex Scientific Ltd, Abington, UK) interfaced to a Varian console (Varian Inc., Palo Alto, CA). An actively decoupled volume transmitter coil and a quadrature surface receiver coil were utilized. Manganese enhanced images were acquired 24 hours after systemic MnCl_2_ injection (intraperitoneal injection, 54 mg/MnCl_2_.4H_2_O/kg in 0.1 M bicine buffer, pH 7.4). T1 weighted 3D MEMRI images were obtained with a gradient echo sequence of repetition time 1200 ms, echo time 4 ms, 70 degrees flip angle, field of view 35 × 35 × 25 mm, matrix 256 × 270 × 64 zerofilled to 256 × 270 × 256 resulting in 137 × 130 × 98 µm resolution.

#### Image analysis

In cohorts 1 and 2, the region of interest (ROI) outlining the hypophysis was manually drawn in the sagittal plane using ImageJ software (National Institute of Health, USA, version 1.51j8) and total hypophyseal volume was assessed. In cohort 2, ROIs were drawn in the coronal plane to estimate the volume of the adenohypophysis (intermediate lobe included) and neurohypophysis separately. Total volume was calculated by multiplying the total number of pixels with the size of each pixel. The data are expressed as mm^3^.

### Pentylenetetrazol (PTZ) test

In cohort 2, continuous video-EEG monitoring was done for all rats after MRI but no spontaneous seizures were detected (see Hayward *et al*., 2010 for details)^29^. In order to assess susceptibility to induced seizures, rats were injected with a single dose (25 mg/kg) of PTZ (1,5-pentamethylenetetrazole, 98%, Sigma-Aldrich YA-Kemia Oy, Finland) at 9 months after TBI or sham operation. Rats were placed in individual transparent plexiglass cages and video-EEG was recorded for 60 min after PTZ administration. An *electrographic seizure* was defined as a >5 sec duration high-amplitude rhythmic discharge with a clear onset, temporal evolution in wave morphology and amplitude, and offset. PTZ-induced epileptiform discharges were defined as a high amplitude rhythmic discharge containing a burst of slow waves, spike-wave and/or polyspike-wave components and lasting <5 sec. A spike was defined as a high-amplitude (twice baseline) sharply contoured waveform with a duration of 20–70 msec. Latency to the first spike, total number of spikes, total number of PTZ-induced epileptiform discharges and total number of electrographic seizures were calculated.

### Morris water-maze

In cohort 2, hippocampus-dependent learning and memory was assessed as previously described using the Morris water-maze^30,31^. Briefly, the test was conducted in a black pool of water (150 cm, 20 ± 2 °C) surrounded by visual cues for animals to orient themselves. The pool was divided into four quadrants, and a platform was placed 1.5 cm below the water in the middle of the northeast quadrant. A video camera connected to a computerized image analysis system (HVS image, Imaging Research Inc., UK) was positioned above the pool. The rats were tested on three consecutive days, with five trials per day on days 1 and 2. The swimming start position was altered in every trial and if a rat failed to find the platform within 60 sec, it was guided to the target manually. Rats were allowed to remain on the platform for 10 sec after each trial. Thereafter, it rested in the cage for 30 sec (after trials 1, 2, and 4) or 1 min (after trials 3 and 5). On day 3, the platform was removed to assess the ability of rats to remember the location of the platform. Latency to the platform (maximum 60 sec), length of the swimming path during the trial (cm), mean swimming speed (cm/sec) and percentage total time spent in each of the four quadrants were measured.

### Statistical analysis

Data was analyzed using IBM SPSS 25.0 for Windows (SPSS Inc., IL, USA). Differences between the sham and TBI groups were analyzed using the Mann-Whitney U-test. Differences between the time points within the sham or TBI groups were analyzed using Wilcoxon’s test. Correlations were assessed using Spearman’s rho correlation coefficient. All data are expressed as mean ± SEM. A p-value of less than 0.05 was considered statistically significant.

## Results

### Hypophysis in MRI

**Figure 1:**
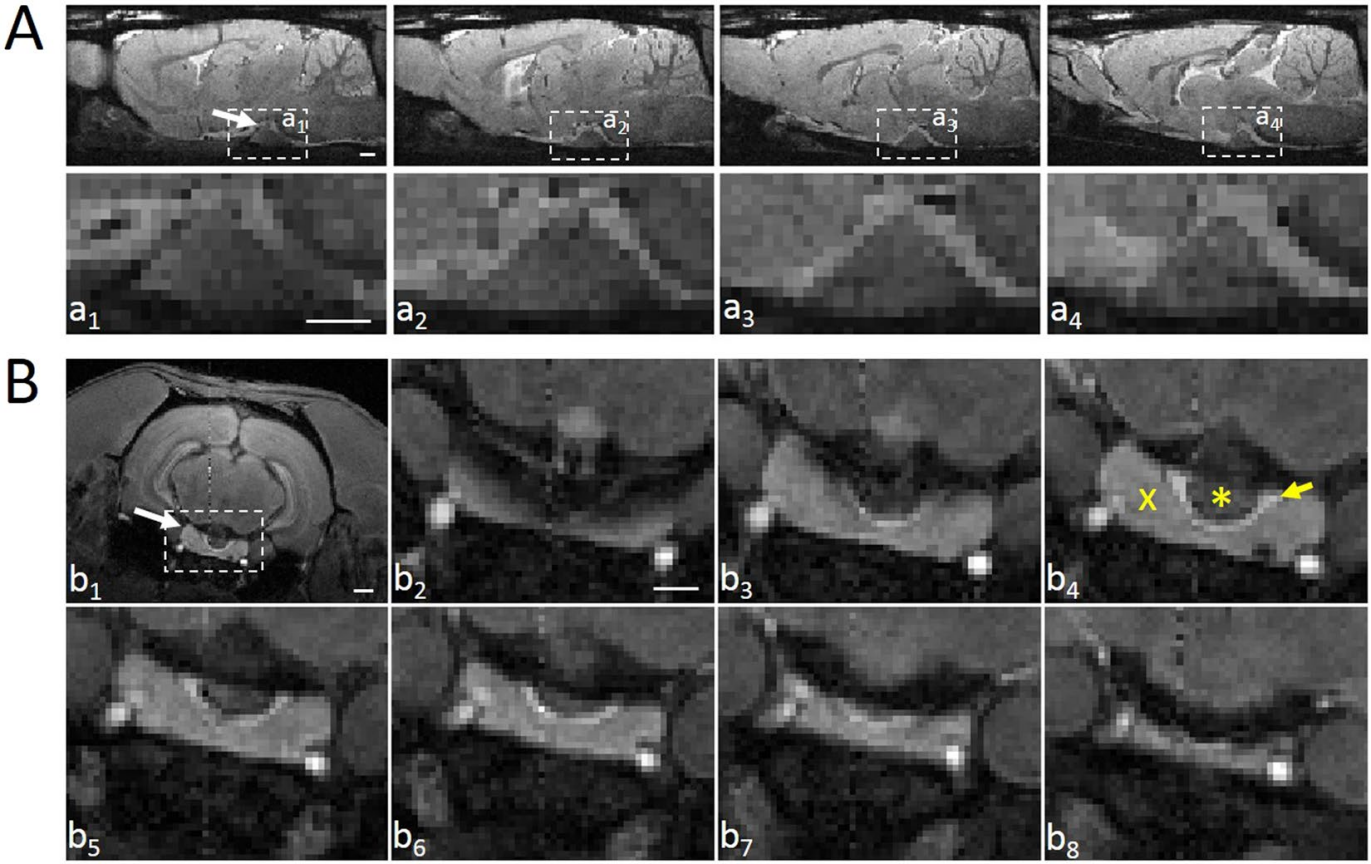
Visualization of the rat hypophysis by MRI and MEMRI in the sagittal and coronal planes, respectively. (**A**) Structural MRI slices (480 µm apart) from a sham-operated rat showing the appearance of the hypophysis (a_1_, white arrow) across sagittal plane. Dashed boxes indicate the area shown in higher magnification (a_1_- a_4_, where a_1_ is the most medial and a_4_ is the most lateral slice). (**B**) MEMRI slices from a sham-operated rat showing the hypophysis (b_1_, white arrow) in coronal plane. The dashed box indicates the area shown in higher magnification in slices (b_2_–b_8_) arranged in the rostral to caudal direction. Note the clear margins separating the adenohypophysis (cross in b_4_), intermediate lobe (yellow arrow in b_4_) and neurohypophysis (asterisk in b_4_). Scale bars equal 1 mm.

Previous MRI studies have demonstrated that the rat hypophysis has an irregular shape and is located on the ventral side of the brain^32^. With MRI, we could identify the outer margins of hypophysis in mid-sagittal (Fig. 1A) and coronal planes but individual lobes could not be distinguished. Therefore, in cohort 2, we employed MEMRI, which allowed us to identify and locate the three lobes of the rat hypophysis (adenohypophysis, intermediate lobe, neurohypophysis) distinctively in the coronal plane^23^ (Fig. 1B).

### Total hypophyseal volume after TBI did not differ from sham-operated experimental controls or show any progression from 2 d to 5 months post-TBI

**Figure 2:**
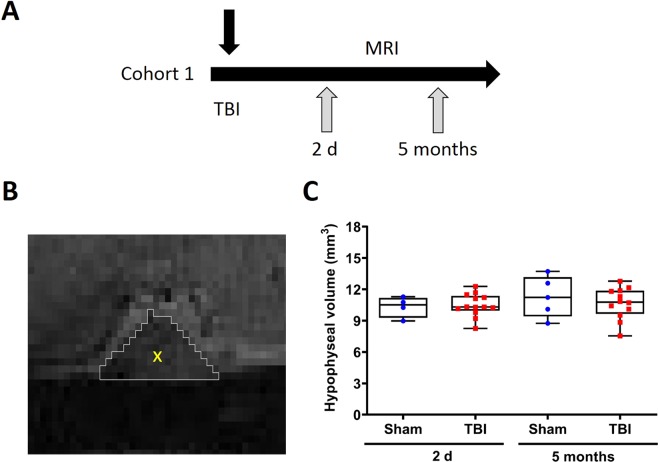
Hypophyseal volume at 2 d and 5 months post-TBI. (**A**) Structural MRI was acquired from sham-operated and injured animals at 2 d and 5 months post-TBI. (**B**) Manually outlined ROI showing the hypophysis in the sagittal plane. **(C)** The total hypophyseal volume did not change at 2 d and 5 months post-TBI when compared to the sham-operated controls. Data is expressed as mean ± SEM.

First, we estimated the total hypophyseal volume in the sagittal plane from structural MRI images of animals scanned at 2 d and 5 months post-TBI (cohort 1) (Fig. 2A,B). The total volume of the hypophysis did not change in the injured group at 2 d post-TBI (10.49 ± 0.23 mm^3^ vs 10.32 ± 0.50 mm^3^) or 5 months post-TBI (10.66 ± 0.43 mm^3^ vs 11.28 ± 0.87 mm^3^) when compared to sham-operated animals (Fig. 2C). There was no change over time in total volume between 2 d and 5 months in controls or in rats with TBI (p > 0.05, Wilcoxon’s test).

### Total hypophyseal volume was not altered in chronic TBI rats at 8 months post-TBI

**Figure 3:**
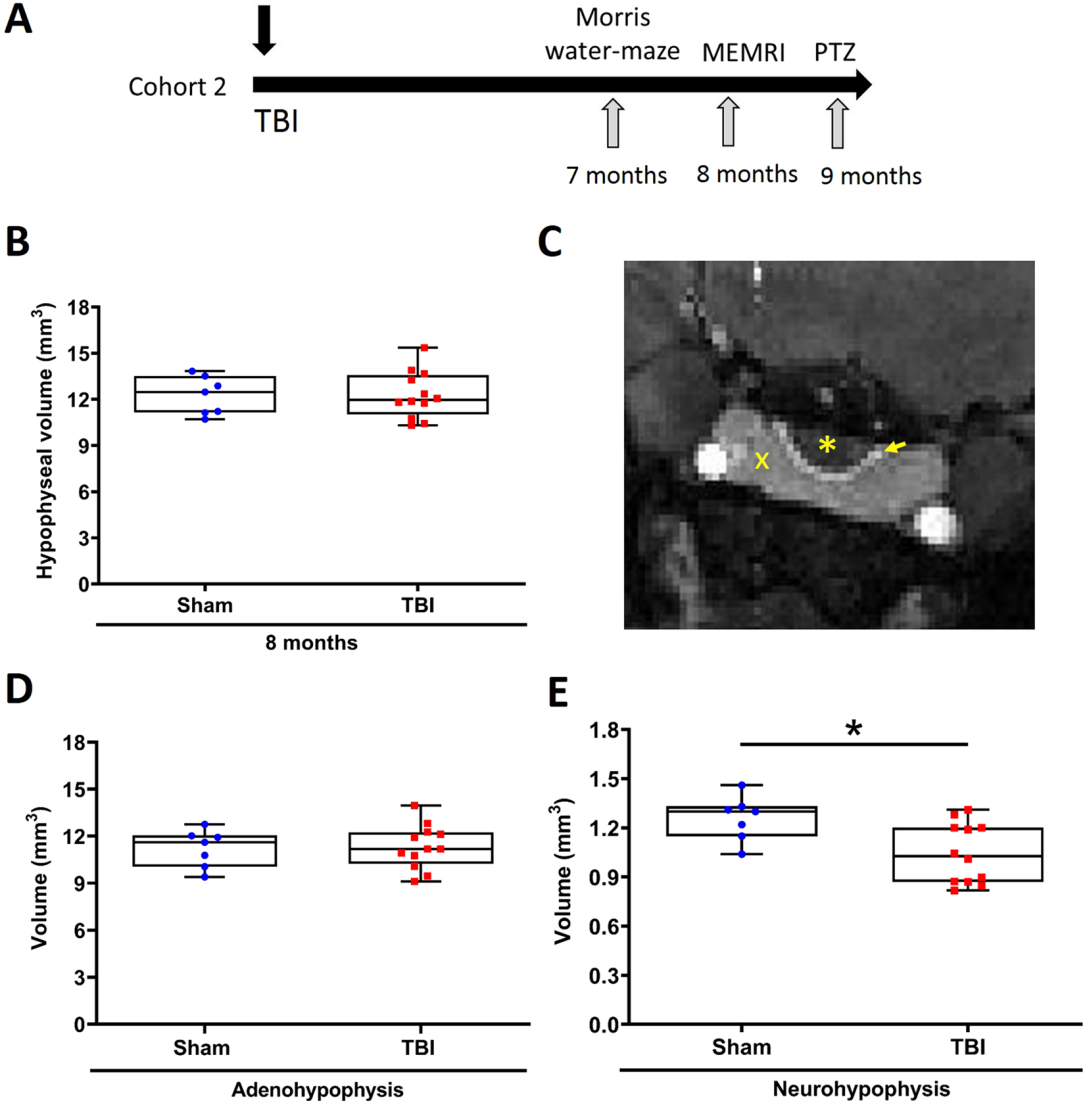
Hypophyseal volume at 8 months post-TBI. (**A**) Study design. MEMRI was performed at 8 months after lateral FPI (Cohort 2). (**B**) The total hypophyseal volume (in the sagittal plane) was unaffected at 8 months post-TBI when compared with sham-operated controls. (**C**) MEMRI showing the posterior (neurohypophysis, yellow asterisk), intermediate (yellow arrow) and anterior (adenohypophysis, yellow cross) lobe in the coronal plane. (**D**) The volume of the adenohypophysis did not differ between TBI and sham-operated controls (p > 0.05). (**E**) The volume of the neurohypophysis was reduced in injured animals as compared to the controls (p = 0.02). Data is expressed as mean ± SEM.

Next, we estimated the total hypophyseal volume in coronal MEMRI images at 8 months post-TBI (cohort 2) (Fig. 3A). The total volume of the hypophysis in the injured group (12.30 ± 0.43 mm^3^ vs 12.25 ± 0.46 mm^3^) was not different when compared to sham-operated animals (Fig. 3B). The total volume of the hypophysis in the sham-operated or injured groups did not differ between 2 d, 5 months and 8 months post-TBI.

### MEMRI indicated reduced neurohypophyseal volume at 8 months post-TBI while adenohypophyseal volume remained unaltered

The hypophysis consists of two lobes; the adenohypophysis (anterior pituitary) and neurohypophysis (posterior pituitary), with their individual distinctive physiological functions^32^. Mn^2+^-induced enhancement of contrast in MEMRI allowed us to define lobe-specific ROIs in the coronal view for lobe-specific volumetry (Fig. 3C). Therefore, in order to identify volumetric changes after TBI in both lobes separately, we calculated the volume of the adenohypophysis and neurohypophysis in sham and injured animals at 8 months post-TBI (Suppl. Fig. 1).

The volume of the adenohypophysis was not different between the TBI and control group (11.32 ± 0.4 mm^3^ vs 11.22 ± 0.45 mm^3^) (Fig. 3D). However, the volume of the neurohypophysis was reduced in injured animals as compared to sham-operated controls (1.04 ± 0.05 mm^3^ vs 1.25 ± 0.05 mm^3^, p < 0.05) (Fig. 3E). Further analysis showed no correlation between the impact severity and the volume of neurohypophysis (r = −0.453, p = 0.140).

Similar total hypophyseal volumes were obtained by volumetric analysis of MEMRI images in both planes. The total hypophyseal volume estimated in the coronal plane was not different from the total hypophyseal volume in the sagittal plane (12.35 ± 0.40 mm^3^ vs 12.29 ± 0.43 mm^3^, p > 0.05) and the volumes correlated with each other (r = 0.97, p < 0.05).

Hypophysis regulates vital body functions including growth and metabolism^33^. Therefore, we investigated whether the total volume of hypophysis, volume of neurohypophysis or volume of adenohypophysis at 8 months post-TBI correlated with the body weight of the rat at the time of MEMRI acquisition. The mean body weight of rats at 8 months post-TBI was comparable with the sham operated controls (578 ± 12 g vs 599 ± 20 g, p > 0.05). The total hypophyseal, adenohypophyseal or neurohypophyseal volume did not correlate with body weight at 8 months post-TBI.

### Total hypophyseal and neurohypophyseal volumes correlate with seizure susceptibility and hippocampus-dependent spatial learning and memory

**Figure 4:**
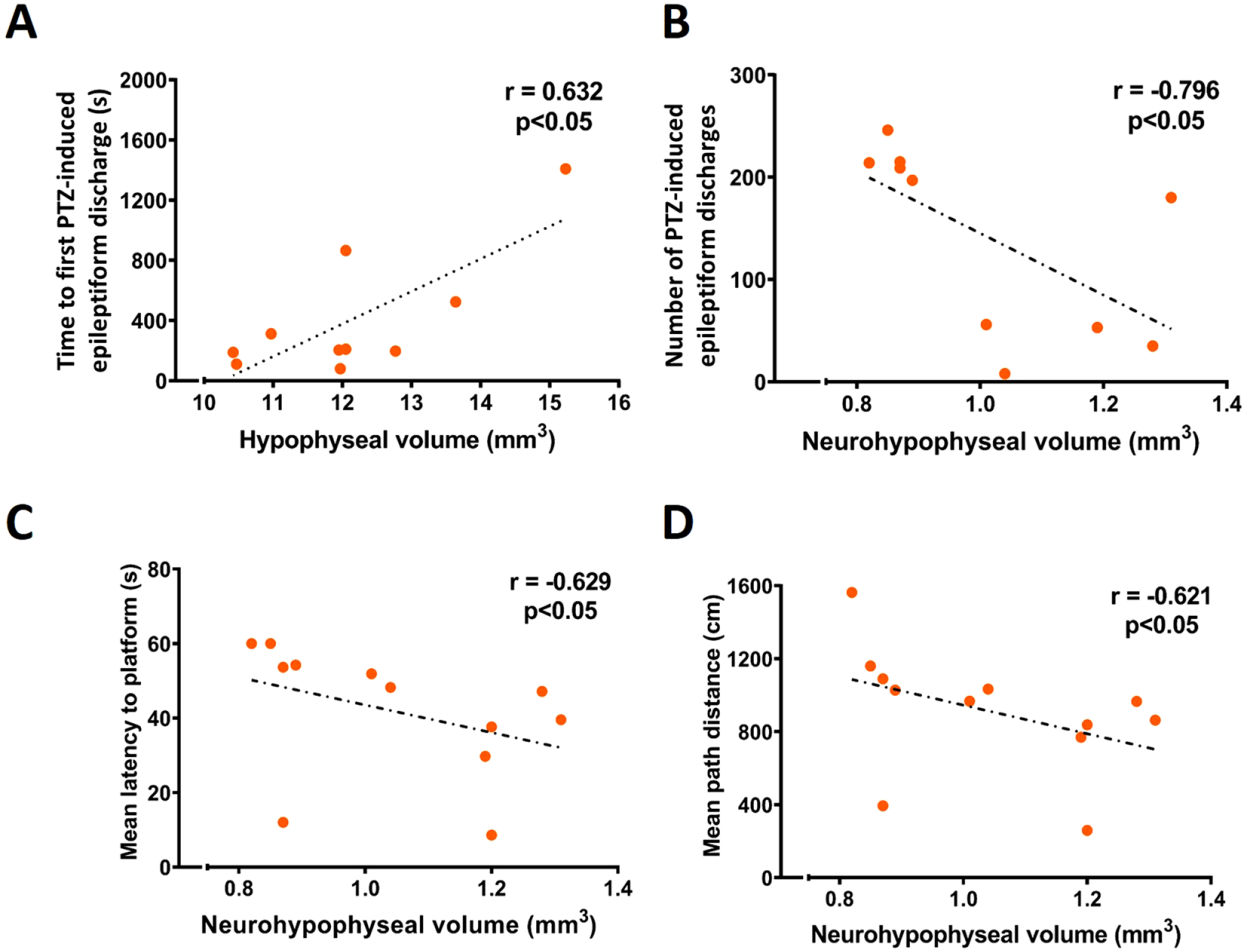
Correlation of hypophyseal and neurohypophyseal volumes with seizure susceptibility and hippocampal-dependent learning and memory. (**A**) The total hypophyseal volume correlated with the time to first PTZ-induced epileptiform discharge. (**B**) The neurohypophyseal volume inversely correlated with the number of PTZ-induced epileptiform discharges in the first 60 min after PTZ injection. The neurohypophyseal volume was also inversely correlated with (**C**) the mean latency and (**D**) the mean path distance in the Morris water-maze.

In order to investigate if hypophyseal volumes are associated with functional outcome after TBI, we analyzed the correlation of hypophyseal, neurohypophyseal and adenohypophyseal volumes with the outcome in the PTZ and Morris water-maze tests. We found that smaller the hypophyseal volume, shorter the time to the first PTZ-induced epileptiform discharge at 9 months post-TBI (r = 0.632, p < 0.05) (Fig. 4A). Smaller the neurohypophyseal volume, greater the total number of PTZ-induced discharges (r = −0.796, p < 0.05) (Fig. 4B). Furthermore, smaller the neurohypophyseal volume, longer the mean latency to platform (r = −0.629, p < 0.05) and mean path distance travelled (r = −0,621, p < 0.05) in the Morris water-maze at 7 months post-TBI (Fig. 4C,D).

## Discussion

Our objective was to investigate whether TBI affects the structure of the hypophysis in rats, and if these TBI-induced structural changes can be detected by MRI and MEMRI. We hypothesized that the hypophysis undergoes structural changes after TBI. We identified a reduction in the volume of the neurohypophysis after TBI using MEMRI. Interestingly, the total hypophyseal volume in rats remained unaltered at both acute and chronic time points after TBI, and the effect of injury could only be detected when the volume of each lobe was investigated individually in the chronic post-injury phase. Small neurohypophyseal volumes in individual animals associated with impaired memory function and increased seizure susceptibility.

The hypophysis is composed of anterior and posterior lobe that have distinct anatomical features and physiological functions^33^. The neurohypophysis receives projections from the paraventricular and supraoptic nuclei of the hypothalamus, and the role of this axis in the regulation of systemic release of oxytocin and vasopressin is well established, in rodents and humans^11^. In humans, a defect in the components of the hypothalamo-neurohypophyseal axis may lead to the development of diabetes insipidus (DI), which is characterized by polydipsia, polyuria, dehydration and hyponatremia^11,34^. Moreover, lesions of hypothalamo-neurohypophyseal axis account for 25% cases of DI in humans and DI occurs in 2.9% of TBI admitted to the intensive care unit^34^.

The etiology of post-TBI pituitary dysfunctions remains unclear so far. However, the anatomical confinement of the pituitary gland in the sella turcica makes it susceptible to damage due to rotational and shearing forces caused by mechanical trauma, and the subsequent hemorrhage can compromise its integrity^9^. The location of the hypophysis in the skull, and its attachment to the hypothalamus, is highly comparable between rodents and humans^9,10^. Therefore, imaging studies in rodents after TBI serve as an appealing source for structural and functional biomarkers for TBI and TBI-associated abnormalities.

Very few studies have used MRI to investigate structural changes in the hypophysis after TBI in humans. A retrospective MRI study in patients with TBI showed an increased hypophyseal volume acutely (<7 days post-injury) that normalized over time (8–15 months post-injury) irrespective of the severity of injury^35^. In our study, we found no difference in the total volume of the hypophysis at 2 d, 5 months and 8 months post-TBI in rats when compared to sham-operated animals. This difference in observation can be attributed to the heterogeneous nature of injury in human cases investigated in Maya *et al*.^35^. Moreover, even though 2 of the 15 chronically followed-up cases showed signs of adenohypophyseal atrophy, lobe-specific volumetric changes were not investigated^35^. Furthermore, no correlation was found between the Glasgow Coma Score (GCS) (a marker of TBI severity) and pituitary volume^35^. Similarly, in our study no correlation was found between impact severity and the volume of neurohypophysis.

MEMRI has provided remarkably detailed delineation of boundaries of the pituitary lobes especially in the coronal plane^22,23^. When we investigated the volume of the adenohypophysis and neurohypophysis individually using MEMRI, we identified that the size of the neurohypophysis is reduced in injured animals at 8 months post-TBI. However, this change did not reflect in the total volume estimates from the same animals, both in coronal and sagittal planes. We did not observe any increase in the volume of the adenohypophysis that could have explained unaltered total hypophyseal volume (Suppl. Fig. 1). It can be suggested that the change in the neurohypophyseal volume, comprising about 8% of the total hypophyseal volume, is masked in the total hypophyseal volume and not detectable due to the relatively large size of the adenohypophysis. Future histologic studies are needed to specify the pathologies leading to pituitary atrophy in this experimental model of TBI.

The total volume of the hypophysis estimated using MEMRI was not different from that using structural MRI. Furthermore, the total volume of the hypophysis in our study was comparable with volumetric estimates in other MEMRI studies using the same strain of rats^36^. The use of MEMRI in humans is not safe due to the cytotoxic effects of Mn^2+^ but it can still serve as a good preclinical tool for investigating post-TBI structural changes when delivered in non-toxic concentrations^23^.

We also noted that TBI animals with a small total hypophyseal volume had a shortened delay to the first PTZ-induced epileptiform discharge, and rats with a small neurohypophyseal volume showed an increased number of discharges after PTZ injection. These findings indicate that pituitary damage is associated with increased seizure susceptibility. It is important to emphasize that aberrant neuroendocrine secretions have been previously noticed as risk factors for seizures in humans^17,18^. For instance, abnormal plasma vasopressin levels lead to hyponatremia due to the syndrome of inappropriate secretion of antidiuretic hormone (SIADH) resulting in increased intracranial pressure and seizures^18^. The neurohypophyseal volume also correlated inversely with mean latency to platform and mean path distance in Morris water-maze test for memory, suggesting that greater the pituitary damage, the poorer the memory performance. Memory impairment is well documented in this animal model of injury and has multiple causes, including hippocampal atrophy and diffuse axonal injury of hippocampal network^37^. Therefore, it can suggested that lobe-specific volumetric analysis of pituitary gland after TBI can serve as a surrogate marker of post-traumatic outcome and evolution of comorbidities. Further investigations are needed to evaluate whether the observed changes in neurohypophyseal volume and their correlations indicate causation or consequence of TBI-associated pathology and comorbidities.

## Conclusions

TBI leads to chronic neurohypophyseal atrophy in rats with lateral fluid-percussion injury. In addition to an overall hypophyseal volumetric analysis, it is important to investigate lobe-specific changes in pituitary structure after TBI. This is particularly important because both lobes have unique functions in body homeostasis. Further studies are needed to explore abnormalities in the hypothalamo-pituitary axis in TBI and PTE, and their mechanistic link with associated comorbidities.

## Supplementary information

Supplementary Information

## Data Availability

The datasets generated during and/or analyzed during the current study are available from the corresponding author on reasonable request.
