# Supplementary material for: Lateral fluid-percussion injury leads to pituitary atrophy in rats
Source: Sci Rep. 2019 Aug 14;9:11819. doi: 10.1038/s41598-019-48404-w (PMC6694150; doi:10.1038/s41598-019-48404-w)
Supplement: Supplementary file 1 — Supplementary Information [file 41598_2019_48404_MOESM1_ESM.docx]

**Lateral fluid-percussion injury leads to pituitary atrophy in rats**

Mehwish Anwer^a^, Riikka Immonen^a^, Nick MEA Hayward^a^, Xavier Ekolle Ndode-Ekane^a^, Noora Puhakka^a^, Olli Gröhn^a^, Asla Pitkänen^a*^

^a^*A. I. Virtanen Institute for Molecular Sciences, University of Eastern Finland, Kuopio, Finland.*

***Corresponding author:** Asla Pitkänen, MD, PhD, A. I. Virtanen Institute for Molecular Sciences, University of Eastern Finland, PO Box 1627, FI-70211 Kuopio, Finland, Tel: +358-50-517 2091, Fax: +358-17-16 3030, E-mail: [asla.pitkanen@uef.fi](mailto:asla.pitkanen@uef.fi)

**
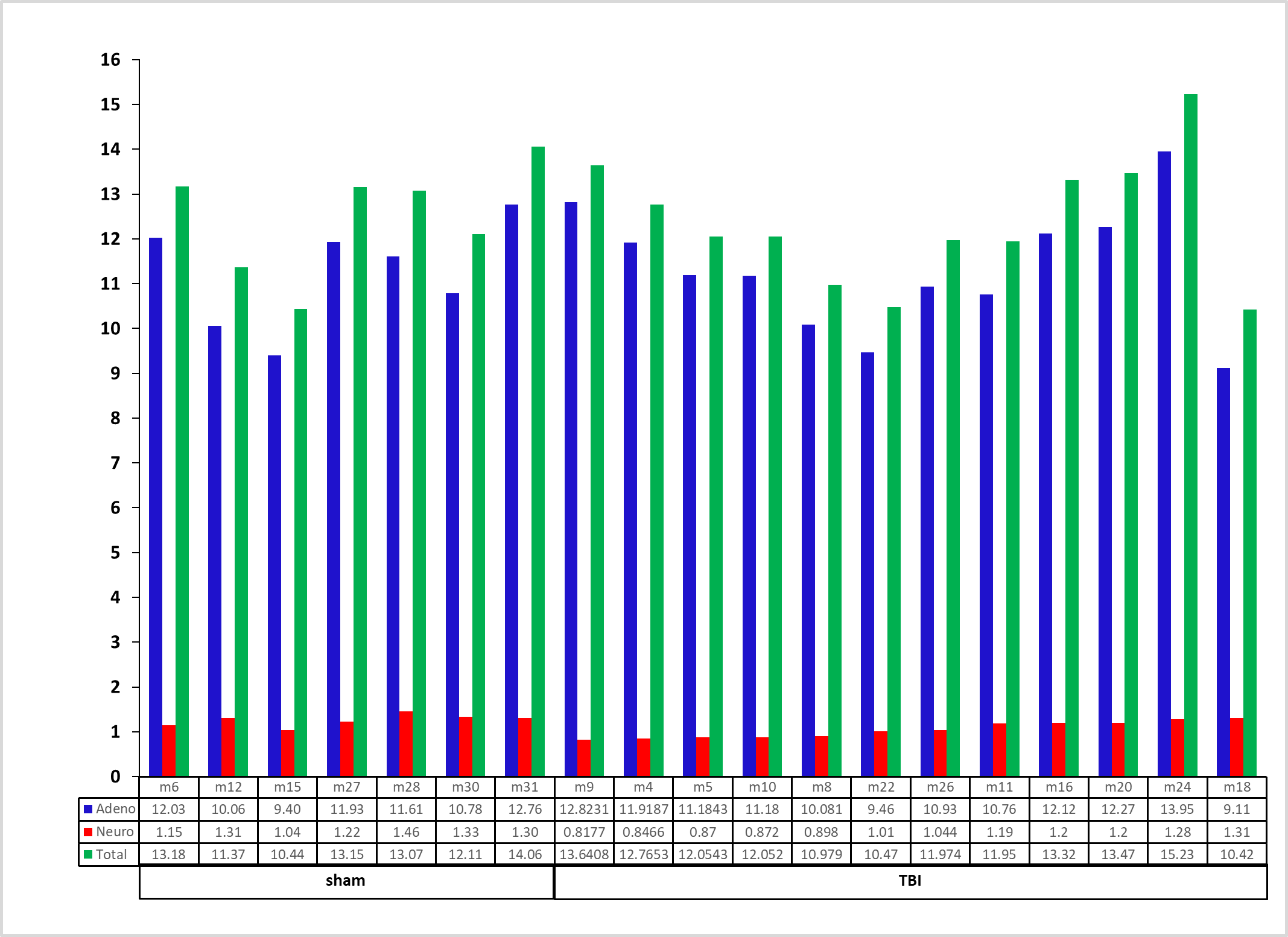
**

**Supplementary Figure 1.** Graph depicting volume of adenohypophysis, neurohypophysis and total hypophysis for all rats investigated at 8 months post-TBI using MEMRI.
